# Management of hand osteoarthritis: from an US evidence-based medicine guideline to a European patient-centric approach

**DOI:** 10.1007/s40520-022-02176-y

**Published:** 2022-07-21

**Authors:** Nicholas Fuggle, Nathalie Bere, Olivier Bruyère, Mario Manuel Rosa, María Concepción Prieto Yerro, Elaine Dennison, Fitnat Dincer, Cem Gabay, Ida K. Haugen, Gabriel Herrero-Beaumont, Mickaël Hiligsmann, Marc C. Hochberg, Andrea Laslop, Radmila Matijevic, Emmanuel Maheu, Alberto Migliore, Jean-Pierre Pelletier, Régis Pierre Radermecker, François Rannou, Brigitte Uebelhart, Daniel Uebelhart, Nicola Veronese, Mila Vlaskovska, René Rizzoli, Ali Mobasheri, Cyrus Cooper, Jean-Yves Reginster

**Affiliations:** 1 MRC Lifecourse Epidemiology Centre, University of Southampton, Tremona Road, Southampton, SO16 6YD UK; 2 European Medicines Agency, Amsterdam, The Netherlands; 3 Division of Public Health, Epidemiology and Health Economics, WHO Collaborating Center for Public Health Aspects of Musculo-Skeletal Health and Ageing, University of Liège, Avenue Hippocrate 13, CHU B23, 4000 Liege, Belgium; 4 Faculdade de Medicina da Universidade de Lisboa, Lisbon, Portugal; 5 Agencia Española de Medicamentos y Productos Sanitarios, Madrid, Spain; 6 Department of Physical and Rehabilitation Medicine, Faculty of Medicine, Hacettepe University, Ankara, Turkey; 7 Division of Rheumatology, University Hospital of Geneva, and Department of Pathology and Immunology, University of Geneva of Medicine, Geneva, Switzerland; 8 Division of Rheumatology and Research, Diakonhjemmet Hospital, Oslo, Norway; 9 Head of Rheumatology Department, Bone and Joint Research Unit, IIS-Fundación Jiménez Díaz UAM, 28040 Madrid, Spain; 10 Department of Health Services Research, CAPHRI Care and Public Health Research Institute, Maastricht University, 6200 MD Maastricht, The Netherlands; 11 Departments of Medicine and Epidemiology and Public Health, University of Maryland School of Medicine, Baltimore, MD USA; 12 Scientific Office, Federal Office for Safety in Health Care, Vienna, Austria; 13 Faculty of Medicine, University of Novi Sad, Novi Sad, Serbia; 14 Clinical Center of Vojvodina, Clinic for Orthopedic Surgery, Novi Sad, Serbia; 15 Rheumatology Department, Hospital Saint-Antoine, AP-HP, and Private Office, Paris, France; 16 Rheumatology Unit - San Pietro Fatebenefratelli Hospital, Rome, Italy; 17 Osteoarthritis Research Unit, University of Montreal Hospital Research Centre (CRCHUM) and Arthritis Division, University of Montreal Hospital Centre (CHUM), Montreal, Canada; 18 Department of Diabetes, Nutrition and Metabolic Disorders, Clinical Pharmacology, University of Liège, CHU de Liège, Liège, Belgium; 19 Service de Rééducation et de Réadaptation de l’Appareil Locomoteur et des Pathologies du Rachis, Hôpitaux Universitaires-Paris Centre, Groupe Hospitalier Cochin, Assistance Publique-Hôpitaux de Paris, Université de Paris, INSERM U1124, Paris, France; 20 Division of Bone Diseases, Geneva University Hospitals and Faculty of Medicine, University of Geneva, Geneva, Switzerland; 21 Division of Musculoskeletal, Internal Medicine and Oncological Rehabilitation, Leukerbad Clinic -Private Rehabilitation Clinic, 3954 Leukerbad, Switzerland; 22 Department of Internal Medicine, Geriatrics Section, University of Palermo, Palermo, Italy; 23 Medical Faculty, Department of Pharmacology and Toxicology, Medical University Sofia, Sofia, Bulgaria; 24 Research Unit of Medical Imaging, Physics and Technology, Faculty of Medicine, University of Oulu, 90014 Oulu, Finland; 25 Department of Regenerative Medicine, State Research Institute Centre for Innovative Medicine, 08406 Vilnius, Lithuania; 26 Departments of Orthopedics, Rheumatology and Clinical Immunology, University Medical Center Utrecht, 3584 CX Utrecht, The Netherlands; 27 Department of Joint Surgery, The First Affiliated Hospital of Sun Yat-Sen University, Guangzhou, 510080 China

**Keywords:** Hand, Osteoarthritis, Management, Patient-centered, Treatment guideline

## Abstract

Hand osteoarthritis is the most common joint condition and is associated with significant morbidity. It is of paramount importance that patients are thoroughly assessed and examined when complaining of hand stiffness, pain, deformity or disability and that the patient’s concerns and expectations are addressed by the healthcare professional. In 2019 the American College of Rheumatology and Arthritis Foundation (ACR/AF) produced guidelines which included recommendations for the treatment of hand osteoarthritis. An ESCEO expert working group (including patients) was convened and composed this paper with the aim to assess whether these guidelines were appropriate for the treatment of hand osteoarthritis therapy in Europe and whether they met with the ESCEO patient-centered approach. Indeed, patients are the key stakeholders in healthcare and eliciting the patient’s preference is vital in the context of an individual consultation but also for informing research and policy-making. The patients involved in this working group emphasised the often-neglected area of aesthetic changes in hand osteoarthritis, importance of developing pharmacological therapies which can alleviate pain and disability and the need of the freedom to choose which approach (out of pharmacological, surgical or non-pharmacological) they wished to pursue. Following robust appraisal, it was recommended that the ACR/AF guidelines were suitable for a European context (as described within the body of the manuscript) and it was emphasised that patient preferences are key to the success of individual consultations, future research and future policy-making.

## Introduction

Hand osteoarthritis is a highly prevalent disease [1] and is associated with substantial morbidity [2, 3]. It can be diagnosed radiographically or clinically with the percentage of radiographic osteoarthritis in the population ranging between 43% (from a global systematic review of patients aged 18–99 years) [4] to an age-standardized prevalence of 44% in females and 37% in males (aged 28–92 years) in the United States [1]. In Europe there are an estimated 748 million inhabitants [5] and the proportion of those aged over 60 years is set to rise [6]. Given osteoarthritis is strongly associated with ageing it is likely that the prevalence of hand osteoarthritis will grow [7]. However, it is important to note that osteoarthritis is a verified disease in its own right and not merely a sequela of a generic ageing process, and so requires specific diagnosis and management. Indeed, the management of hand osteoarthritis should be multimodal including pharmacological and non-pharmacological approaches as documented in the recommendations from international learned bodies [8–10].

The American College of Rheumatology (ACR) and the Arthritis Foundation (AF) have recently published guidelines for the management of hand osteoarthritis [9]. In 2021 ESCEO (The European Society for Clinical and Economic Aspects of Osteoporosis, Osteoarthritis and Musculoskeletal Diseases) convened an expert working group which comprised patients, clinicians, researchers, economists and regulators who performed reviews of the current literature relating to the management and patient preferences research in hand osteoarthritis. The primary aim of this group was to appraise (within the context of the current literature) whether these US guidelines were appropriate for a European context and to highlight the importance of a patient-centered approach to hand osteoarthritis management.

## Heterogeneity of hand osteoarthritis

Hand osteoarthritis is an umbrella term under which sits an increasing number of clinical phenotypes [11]. Possible risk factors of the condition include; age-related, genetic, inflammatory (complement proteins, inflammageing, innate immunity and systemic mediators) and metabolic (encompassing obesity and diabetes mellitus and possible cardiovascular associations) [12, 13].

These manifold causes are of paramount clinical importance when assessing a patient with hand pain. It is crucial to determine the types of symptoms, the onset of symptoms, the pattern of disease flares, extent of deformity (both soft tissue and bony enlargement) and the comorbid context of the condition, as these factors allow robust clinical decision-making with regard to diagnosis and management.

The clinical complaints which lead patients to approach healthcare services include pain, stiffness, loss of function, reduced quality of life, worries about the future and aesthetic concerns related to deformity [14]. The latter two of these are particularly prevalent in younger patients with osteoarthritis (especially women), and, radiographic assessment is highly informative to ascertain the trajectory of progression.

Hand radiographs are often used to confirm the diagnosis of hand osteoarthritis, to assess the degree of structural changes and to document the distribution of joint involvement. This latter point is particularly relevant given the different phenotypes of osteoarthritis affecting the interphalangeal joints or the thumb base joints [15]. The extent of damage for osteoarthritis can be graded as none, doubtful, definite, moderate and severe according to Kellgren and Lawrence radiographic scoring [16]. Erosive osteoarthritis is a particular phenotype which is characterised by central subchondral bone collapse and is associated with greater pain, a higher level of disability [3] and even an inflammatory element [17, 18]. Indeed, inflammatory osteoarthritis (which can occur independently of erosive osteoarthritis) is observed as increased grey scale synovitis on ultrasound (indicating synovial thickening and/or effusion) and even, occasionally, associated with increased synovial blood flow on power doppler examination [19]. Erosive osteoarthritis can be graded as normal, stationary, destructive or remodelling, according to the radiographic appearances [20].

After the clinical and/or radiographic examination has been performed, a management plan is then devised together with the patient once the patients’ preferences have been elicited.

## The opinion of patients

The ESCEO expert working group included patients and their contribution provided novel insights into patient preferences and perspectives. They emphasised that hand osteoarthritis was a highly debilitating disorder with substantial impacts on function (“holding more than one kilogram was too heavy”), the common activities of daily living (“I was unable to turn a key”) and independence (“asking for help was very unpleasant”). They also experienced severe symptoms (“pain like hot oil on your skin”) which occurred with a clear cyclical pattern with inflammatory flares followed by relief and they also drew attention to the often-forgotten aesthetic concerns which arise due to finger deformations in hand osteoarthritis.

One of the patients described an encouraging response to celecoxib and chondroitin sulphate with, “a rapid response to medication”, however, she halted the celecoxib due to, “a fear of side effects” she had read about in the lay press. This emphasises how important it is to have open channels of communication between patients and clinicians to address these kinds of potential safety concerns and improve adherence.

Another patient had little response to pharmacological approaches and opted for surgical intervention and has been very satisfied with the result. This highlights the importance of communicating all available management options to patients as their disease progresses.

All patients agreed that pharmacological therapies should focus on alleviating pain and dysfunction and be combined with a beneficial safety profile. In some healthcare systems economic considerations are also taken into account including costs of medication and non-pharmacological therapies (such as rehabilitation and physical therapy), particularly if the patient is funding their own care.

With regard to intra-articular therapies (including hyaluronic acid and corticosteroids), all patients agreed that the size of the needle and volume of injection should be minimised, and the interval between injections should be as long as possible.

Conversations around these topics (specific symptoms for the patient, pharmacological/surgical intervention, the desired outcomes of an intervention, tolerability of intra-articular therapy for the individual patient) should be a mainstay when eliciting patient preferences.

## Patient preferences and shared decision-making

ESCEO is a patient-centered organisation which aims to promote the use of patient perspectives in research and intervention development, policy-making and to ensure that patients are encouraged to take an active role in the clinical decision-making surrounding their individual care [21].

When it comes to deciding on a course of management in a clinical setting, the clinician’s choice and the patient’s choice intersect at the point at which a partnership is formed through shared decision-making. This is vital in order to elicit the patients’ preferences.

Patient preferences, with regard to medications, have been defined as “a mental process that patients use to compare and choose one drug over another by comparing the good and bad” [22]. These preferences are vital to decision-making for an individual in a clinical setting, but also a population at a policy level.

In the clinical setting, patient preferences help healthcare professionals to tailor the management plan at the patient level, to facilitate true, shared decision-making and to improve patient ‘buy-in’ and adherence to therapy.

At a policy level, patients should play a key role in the design of healthcare interventions and models of care, the population level assessment of risk-benefit (from a regulatory perspective) and the assessment of the value of an intervention (from a reimbursement perspective).

Methods to elicit preferences have been devised in the discipline of health economics and include ‘revealed’ preferences and ‘stated’ preferences. Revealed preferences are observed by recording consumer choice in a free market, however, this is difficult and impractical in a healthcare setting where ‘choice’ may be influenced by local prescribing behaviours, clinician preference and the level of evidence of a therapy.

Stated preferences are based on hypothetical choices and can be established via exploration (in focus groups or structured interviews) or via elicitation using a discrete choice experiment (DCE). A DCE is a tool which decomposes a product into its constituent ‘attributes’ (or characteristics) which are then placed before a group of patients/consumers in the form of a number of discrete (often binary) choices. Statistical analyses are used to produce an economic model which calculates the attributes that are most important to the patients/consumers. The product can then be developed with these preferences in mind.

DCEs have been utilised in an attempt to elicit patient preferences for the management of osteoarthritis. A previous ESCEO DCE of patients with hip and knee osteoarthritis showed that the most important treatment attributes were impact on disease progression followed by the ability to improve walking and reduce pain [23]. In another US DCE study, including hip and knee osteoarthritis patients, healthcare providers and insurance company employees, the most important attribute was out of pocket costs [24] (though it should be noted that this was not included as an attribute in the ESCEO study and might be related to the US healthcare distribution system). Another DCE in the US included patients with osteoarthritis and chronic low back pain and found a preference for daily oral medication over longer term intravenous therapy [25]. The only DCE in hand osteoarthritis was directly related to the question of preference for arthroplasty versus arthrodesis as a surgical intervention and found that post-operative stiffness, grip strength, cost and need for future surgical procedures were all important attributes [26]. Indeed, the only attribute which was not important was ‘recovery time’ and this may have been because the choice was only between 10 week or 12 weeks. Despite the lack of specific hand osteoarthritis DCEs, it is possible to see that this tool could be used to create a holistic impression of patient preferences for therapies in this disease area and should be the subject of future research.

In summary, there is an increasing emphasis on patient-centered research (outcome assessment tools, expectations and therapeutic management) and it is therefore vital to engage patients in identifying current research needs, the design and conduct of clinical studies and subsequent regulatory assessment [21]. They should also be consulted on post-marketing safety surveillance and efficacy data collection and, if applicable, be represented in the learned bodies devising practice guidelines. Indeed, patient preferences should be incorporated throughout the medicinal (and non-pharmacological) product life cycle [27].

It is not enough for patients to sit on panels. They should also be trained and educated to enable them to engage in the most comprehensive way possible and offer insights on the most complex elements of clinical science. Capturing patient perspectives is a highly active process and should incorporate multiple approaches to engagement [28].

Thus, in managing hand osteoarthritis in the clinic, in drawing up a research road map and informing policy for hand osteoarthritis we must involve the patient as the key stake-holder. This will lead to better adoption of interventions, better patient satisfaction and better therapeutic adherence.

## Management of hand osteoarthritis: the ACR/AF 2019 guidelines

In 2019, ESCEO published a treatment algorithm for the management of knee osteoarthritis [29] which has been endorsed beyond Europe in other continents [30, 31]. Although this is not specific to hand osteoarthritis, the key messages and progression through therapies can be transferred as a guide to use in osteoarthritis of the hand. Other European recommendations for the management of hand osteoarthritis have been published, but without a patient-centered focus or algorithm for therapeutic escalation [32]. In 2019, the ACR and AF published evidence-based recommendations for the management of hand osteoarthritis, which may be used to inform practice in Europe [9].

In this guideline, hand osteoarthritis is defined according to ACR classification criteria [33]. These include the presence of hand symptoms (limited to pain, aching, stiffness) and the findings of clinical examination [33]. Three key features are elicited from specific examination of the distal and proximal interphalangeal joints of the 2nd and 3rd fingers and the trapezio-metacarpal joints of both hands (10 joints). These features include: hard tissue enlargement of ≥ 2 of these ten joints or ≥ 2 distal interphalangeal joints or deformity of ≥ 1 of these joints. In addition to the above, there must be fewer than three swollen metacarpophalangeal joints. If three or four of the above features (in addition to the presence of hand symptoms) are identified then this confers a clinical diagnosis of hand osteoarthritis.

The authors of the ACR/AF 2019 recommendations [9] used a robust methodology in which a core committee of 5 experts honed the scope of the literature review with regard to population, indication, comparator group and outcomes (PICO). The outcomes were defined as improvement in pain and improvement in function. Literature searches were performed up to and including August 2018 and a meta-analysis was performed for the collated papers.

A wider community of experts, including patient representatives, then voted on the evidence for each hand osteoarthritis intervention, and graded them as either a ‘strong’ or ‘conditional’ recommendation ‘for’ or ‘against’ the intervention. A patient panel was also formed as part of the guideline formulating team.

A recommendation was described as ‘strong’ if greater than or equal to 70% of the constitutive committee voted either for or against the use of the intervention and a ‘conditional’ recommendation was made if 50–70% of the committee voted for or against the intervention.

A strong recommendation ‘for’ an intervention meant that patients should receive the intervention for hand osteoarthritis, a strong recommendation ‘against’ an intervention meant that patients should not receive the intervention for hand osteoarthritis. A conditional recommendation meant that a process of shared decision-making (between patient and clinician) should be used to decide if the patient was to receive the intervention.

Interventions were grouped into two categories ‘physical, psychosocial and mind-body’ (avoiding the term ‘non-pharmacological’) and ‘pharmacological’. In the first of these, strong recommendations were made for the use of self-efficacy programmes, self-management programmes and 1^st^ carpo-metacarpal joint orthoses. Conditional recommendations were made for the use of cognitive behavioural therapy, acupuncture (complementary medicine), heat therapies (including paraffin wax baths) and orthoses for the interphalangeal joints.

For pharmacological agents, a strong recommendation was made for oral non-steroidal anti-inflammatory drugs (NSAIDs) and conditional recommendations for topical NSAIDS, intra-articular injections of glucocorticoid, chondroitin sulphate, paracetamol, duloxetine and tramadol (the latter three inferred from knee osteoarthritis studies, in the absence of hand osteoarthritis-specific literature).

Strong recommendations were made against the use of glucosamine (hydrochloride and sulphate), hydroxychloroquine, methotrexate, anti-TNF and IL-1 inhibitors. Conditional recommendations were made against intra-articular injection of hyaluronic acid and the use of topical capsaicin.

In light of these guidelines we will document some of the key evidence for pharmacological therapies in hand osteoarthritis and highlight the non-pharmacological recommendations from the ACR/AF 2019 guidelines. This is followed by a summary of potential novel interventions for hand osteoarthritis.

## NSAIDs and COX-2 inhibitors

These medications have been in usage for the treatment of osteoarthritis for decades and their efficacy (in osteoarthritis though perhaps not hand-specific disease) and safety profiles are well-established (with documented adverse effects including GI complications, cardiovascular events, renal impairment and hypersensitivity, headaches, dizziness, rarely hepatotoxicity, drug interactions) [34].

Non-selective NSAIDs are well known to be associated with upper gastrointestinal, renal and cardiovascular adverse effects. A large meta-analysis demonstrated that the relative risk (RR) of upper gastrointestinal adverse events with naproxen (RR 4.22 (95% CI 2.71, 6.56)) was marginally (numerically) higher than ibuprofen (RR 3.97 (95% CI 2.22, 7.10)) and substantially higher than diclofenac (RR 1.89 (95% CI 1.16, 3.09) [35]. The use of a proton pump inhibitor contemporaneously to NSAIDs reduces the risk of upper GI adverse events to the same level as a COX-2 selective formulation [36].

Cardiovascular adverse effects of NSAIDs have been the subject of extensive investigation with a comparison of agents showing that a significantly increased risk is observed with naproxen (RR 1.87 (95% CI 1.10, 3.16)) and diclofenac (RR 1.85 (95% CI 1.77, 2.94)) but not with ibuprofen (RR 1.44 (95% CI 0.89, 2.33)) [35]. A more recent meta-analysis included 26 randomised controlled trials of non-selective NSAIDs and COX-2 inhibitors which provides a granular assessment of cardiovascular risk and showed that rofecoxib was the only agent with an increased odds of myocardial infarction (OR 1.81 (1.38, 2.38)) and composite cardiovascular risk score (OR 1.6 (95% CI 1.31, 1.98)) [37]. Celecoxib was associated with reduced odds of stroke and cardiovascular adverse events (though there was substantial heterogeneity in the doses of celecoxib used in the meta-analysis) [37]. This was supported by the safety outcomes of the PRECISION trial which demonstrated that celecoxib was non-inferior to both ibuprofen and naproxen in terms of cardiovascular risk [38]. Cardiovascular and gastro-intestinal risks seem to increase with age, but recent data did not show an age-related increase of the RR. Rather, it seems that there is an age-related increase of the absolute risk, multiplied by around 2 for either CV or GI risks between 70–79 and ≥ 80 years old [34]. Patients should be counselled using this information prior to prescription.

Analyses performed by an ESCEO working group investigated the adverse effect profiles of pharmaceutical interventions in osteoarthritis. For COX-2 inhibitors, meta-analyses showed marginally increased risks of abdominal pain (Relative Risk (RR) 1.40 (95% CI 1.08, 1.80)), hypertension (RR 1.45 (95% CI 1.01, 2.10), rofecoxib only), peripheral oedema (RR 1.61 (95% CI 1.09, 2.40)) and generalised oedema (RR 1.91 (95% CI 1.08, 3.39)) compared to placebo [39].

Topical agents were associated with an increased risk of any adverse event (RR 1.16 (95% CI 1.04, 1.29)) and withdrawal due to an adverse effect (RR 1.49 (95% CI 1.15, 1.92)) (which was largely driven by the effect of topical diclofenac) [40], but the risk of serious adverse events was not significantly increased (RR 0.79, (95% CI 0.37, 1.71)).

Although the above studies did not focus specifically on hand osteoarthritis, they do depict the therapeutic and safety landscape of non-selective and selective NSAIDs in osteoarthritis in general and justify their inclusion as strong recommendations in the ACR/AF 2019 guidelines [9].

## Oral paracetamol

Paracetamol, which was only conditionally recommended in the ACR/AF 2019 guidelines[9], provides an analgesic benefit (standard mean difference of pain score between treatment versus placebo group) of 0.14 (95% CI 0.05, 0.22) in knee osteoarthritis [41, 42], though the overall mortality relative risk is 1.28 (95% CI 1.26, 1.30) [43]. Based on these data the true risk of paracetamol may well be higher than currently perceived in the clinical community and, thus, paracetamol should only be used for short-term relief, if at all.

## SYSADOAs

The Symptomatic Slow-Acting Drugs for Osteoarthritis (SYSADOAs) include glucosamine sulphate, chondroitin sulphate and diacerein which aim to alleviate symptoms and elicit functional improvement [44–46]. Meta-analyses have demonstrated small to moderate benefit for prescription-grade crystalline glucosamine sulphate, chondroitin sulphate and diacerein in osteoarthritis it general [44, 47–49] and a 2019 meta-analyses of safety found no substantial adverse effects from this class of medications [50].

Hand-specific osteoarthritis studies include a retrospective, observational study of glucosamine sulphate in 108 participants which demonstrated a significant reduction in pain visual-analogue score and Functional Index of Hand Osteoarthritis (FIHOA) score against control at both 3 and 6 months of follow-up [51]. Contrary to this demonstration of symptomatic benefit, a randomised controlled trial of diacerein in 86 participants in 2013 showed no change in hand pain as measured by the Australian/Canadian hand index (AUSCAN) pain score, though this was only over 4 weeks of follow-up [52].

The role that these agents play in the treatment of osteoarthritis differs between learned bodies with ESCEO recommending pharmaceutical-grade glucosamine sulphate and/or chondroitin sulphate as first-line background treatments but OARSI strongly recommending against their use in hip and knee osteoarthritis [53]. In the ACR/AF 2019 guideline, chondroitin sulphate is conditionally recommended for the treatment of hand osteoarthritis (the conditional nature of this recommendation being due to the small evidence base) [9] which aligns with the EULAR recommendations from 2018 [32].

Chondroitin sulphate is a complex sugar found in the cartilage of some animals and fish and is taken as dietary supplement in order to stimulate cartilage repair and reduce cartilage degradation. There are data to support the use of chondroitin sulphate in osteoarthritis [46] (including beneficial effects on symptoms of up to 3 months [54]) but fewer relating specifically to hand osteoarthritis. Structural improvements including reduced structural damage and reduced occurrence of erosive hand osteoarthritis have previously been demonstrated [55].

Gabay and colleagues performed a well-powered, randomised controlled trial of chondroitin sulphate which included 162 participants (80 chondroitin sulphate and 82 placebo) and 6 months of follow-up. For the primary outcomes there was an approximately 20 mm reduction in visual analogue score for pain, a reduction in FIHOA score (of approximately 3 units, where 30 is the worst possible score) and (for secondary outcomes) a reduction in the duration of early morning stiffness (by approximately 5 min) for those treated with chondroitin sulphate compared to the placebo group [56]. Although no effect was observed on grip strength, rescue paracetamol usage or safety, the symptomatic benefit of chondroitin sulphate, combined with good tolerance, justify its inclusion in the ACR/AF guideline[9] as a conditional recommendation for hand osteoarthritis.

It is recognised, from a scoping review by Honvo and colleagues [57] on the role of collagen derivatives in osteoarthritis, that further research is needed to make definitive conclusions regarding the role of SYSADOAs in the treatment of osteoarthritis as a whole and hand osteoarthritis as a specific case.

## Intra-articular injections

In the 2019 ACR/AF guideline for osteoarthritis management [9], intra-articular injection with corticosteroid is conditionally recommended and injection with hyaluronic acid is conditionally recommended against.

For the corticosteroid injection recommendation it is important to recognise that the EULAR 2018 guidance [8] is similar in stating that injection can be considered specifically for those with painful interphalangeal joints (through the ACR/AF guidance [9] provides no specific recommendation for the anatomical location (interphalangeal or carpometacarpal) which can be injected).

In the EULAR 2018 guideline[8] it is not recommended to inject the base of the thumb with steroid (hyaluronic acid is not addressed) in hand osteoarthritis, likely based on findings (such as that of Kroon and colleagues[32]) of no clear benefit of corticosteroid or hyaluronic thumb base injections.

However, intra-articular, base of thumb injections of hyaluronic acid [58, 59] (for chronic management) and corticosteroid [59–61] (for acute phase management) appear to be promising approaches and could be conditionally proposed (though with the caveat that further efficacy and safety data are required).

## Physical, psychosocial and mind–body approaches

There are a host of non-pharmacological interventions considered for hand osteoarthritis. Exercise is strongly recommended with a focus on overcoming “barriers to participation” by addressing “patient preference and access” (access referring to the affordability and transportation to attend the given exercise intervention [9]. Weight loss is strongly recommended for hip and knee osteoarthritis but not for hand osteoarthritis) [9]. The recommendation for exercise in hand osteoarthritis is supported by a Cochrane review from 2017 which stated benefits such as reduced pain and stiffness [62]. However, the type of exercise to perform, the duration, number and rhythm of sessions need to be precisely specified to patients who frequently ask for this kind of information. Interventions which are recommended for ‘consideration’ in the management of hand osteoarthritis in the ACR/AF 2019 guideline include orthoses (particularly for thumb base OA, but also including digital orthoses, ring splints), cognitive behavioural therapy, thermal interventions, paraffin baths, kinesiotaping for the 1^st^ CMC joint and acupuncture [9]. Iontophoresis is conditionally recommended against for patients with 1^st^ CMC osteoarthritis due to an absence of randomised controlled trials [9].

The gut microbiome is an area of interest with regard to osteoarthritis symptoms and it is clear that alterations in gut microbiota are associated with osteoarthritis and can alter drug metabolism and bioavailability [63]. Nutritional interventional studies support a potential contribution of the gut microbiome to osteoarthritis in both animal models and human subjects [63].

It is also worth considering and treating concurrent conditions including fibromyalgia which have a substantial prevalence in patients with osteoarthritis [64].

## Novel therapeutic options

There are a number of therapeutic approaches being evaluated or developed for the treatment of hand osteoarthritis. Indeed, at the time of writing, there are 87 studies of hand osteoarthritis on clinicaltrials.gov including 15 ongoing trials of agents such as methotrexate, colchicine and cannabinoids.

A total of 16 non-pharmacological interventions have been trialled including exercise, orthoses, education, mud packs and transcutaneous auricular vagus nerve stimulation. Studied pharmacological agents included topical therapies, SYSADOAs, cannabidiol, pregabalin, apremilast and biologic agents including denosumab, tocilizumab and adalimumab.

Trials of biologic agents have shown no significant benefit to date, including; lutikizumab [65], etanercept [66], otilimab [67], adalimumab [68, 69], tocilizumab [70]. Benefit is yet to be demonstrated for hydroxychloroquine [71–73], colchicine [74] or methotrexate [75] and the recent published trials do not advocate in favour of the use of these drugs.

Oral steroid does appear to provide some benefit for flares of hand osteoarthritis, as observed in the Hand Osteoarthritis Prednisolone Efficacy (HOPE) trial [76]. This was a double-blind, placebo-controlled trial of 10 mg oral prednisolone across two sites in the Netherlands, focused on hand osteoarthritis patients with evidence of active inflammation at the interphalangeal joints. Six weeks of prednisolone (with a 2-week tapering period) led to a reduction in finger pain visual analogue score with a mean between-group difference (for prednisolone vs placebo) of -16.5 (95% CI -26.1, − 6.9, p = 0.0007) (in favour of prednisolone) observed, suggesting a role for a short course of oral prednisolone in treatment of osteoarthritis flares.

GCSB-5, a mixture of 6 herbal extracts, has shown promise in a Korean study which demonstrated some symptomatic benefit in hand osteoarthritis at 4 weeks (sustained to 16 weeks) with a reduction of AUSCAN pain score greater than that seen with placebo (-9.0 (95% CI -23.8, -0.4) vs -2.2 (95% CI -16.7, 6.0), p = 0.01) [77]. There is a relative paucity of literature relating to the potential benefits of alternative therapies (including ginger, curcumin, protein-rich plasma) for osteoarthritis and further high quality research is required [78].

## Conclusion

Hand osteoarthritis is a distinct disease rather than simply a sequela of aging which requires multimodal management in the form of non-pharmacological and pharmacological approaches. Through the review and analysis of this ESCEO working group it is clear that the ACR/AF 2019 guidelines for hand osteoarthritis followed a robust methodology and are formally endorsed by ESCEO for usage for the management of European patients.

ESCEO agrees that, within the SYSADOAs family, chondroitin sulphate is the only agent which has successfully demonstrated efficacy for pain and function (in this particular indication). This evidence of efficacy is complemented by good tolerance and therefore justifies that the positive recommendation from the ACR/AF is applied to the European population. It would, nevertheless, be interesting to see further trials confirming the study by Gabay and colleagues [56] (despite the fact that this study was conducted with a robust methodology and an independent statistical analysis of the outcomes).

The ESCEO guidelines are written using a patient-centric approach and in this particular case, we received two fascinating case-studies from patients who highlighted the patient experience and patient preference. The main point that should be reemphasized is that both patients described hand osteoarthritis as a truly disabling disorder. One of them was very happy with the therapeutic approaches that were offered to her (chondroitin sulphate and Celecoxib) but her Celecoxib therapy was interrupted due to concerns (born of the lay press) regarding the possible cardiovascular adverse effects of coxibs. This highlights that it is immensely important to facilitate proper communication between physicians and patients, since these putative adverse effects have not been confirmed for celecoxib. She clearly expressed a cyclical pattern of pain with periods of inflammation (flares) followed by periods of relief. She also experimented with alternative medicines some of which had had a positive effect.

The second patient also described the serious impact of hand osteoarthritis on her daily life. As opposed to the first patient, she was not content with the pharmacological approaches that were offered to her and decided to pursue surgical intervention (proximal interphalangeal prosthesis). Despite the significant financial implication, she is very satisfied with the surgical outcome.

Both patients insisted that pharmacological approaches should provide alleviation for pain and disability, and should be associated with a good safety profile. They highlighted the fact that they might be prepared to consider paying a financial premium, providing that the medication (or the surgical treatment) was effective. Regarding intra-articular hyaluronic acid or steroid injections, they emphasised that they need to see some proof of efficacy and that they would prefer devices using a small needle, minimal volume of injection and, if possible, as few injections as possible.

Patient preference research and the derived health economics analyses support the use of a pharmacological management of hand osteoarthritis. Corticosteroid injections appear to be a promising approach in the acute phase of the disease with hyaluronic acid for chronic symptoms but both require further demonstrations of efficacy and safety. This is also the case for several medications (including glucosamine sulphate) which were or are currently undergoing investigation. Biologic agents have shown no significant benefit, to date.

In conclusion, this paper describes a patient-centered approach to hand osteoarthritis care and highlights the endorsement of the ACR/AF 2019 hand osteoarthritis guidelines for usage in European patients.
